# A****β**** Damages Learning and Memory in Alzheimer's Disease Rats with Kidney-Yang Deficiency

**DOI:** 10.1155/2012/132829

**Published:** 2012-05-08

**Authors:** Dongmei Qi, Yongfa Qiao, Xin Zhang, Huijuan Yu, Bin Cheng, Haifa Qiao

**Affiliations:** ^1^Neuroscience Program, Shandong University of Traditional Chinese Medicine, Changqing University Park, Jinan 250355, China; ^2^Qingdao Haici Medical Group, 4 Renmin Road, Qingdao 266033, China; ^3^Institute of Acupuncture and Moxibustion, China Academy of Chinese Medical Sciences, 16 Nanxiaojie, Dongzhimeinei, Beijing 100700, China; ^4^Department of Biomedical Sciences, Florida State University College of Medicine, 1115 West Call Street, Tallahassee, FL 32306, USA

## Abstract

Previous studies demonstrated that Alzheimer's disease was considered as the consequence produced by deficiency of Kidney essence. However, the mechanism underlying the symptoms also remains elusive. Here we report that spatial learning and memory, escape, and swimming capacities were damaged significantly in Kidney-yang deficiency rats. Indeed, both hippocampal A**β**_40_ and 42 increases in Kidney-yang deficiency contribute to the learning and memory impairments. Specifically, damage of synaptic plasticity is involved in the learning and memory impairment of Kidney-yang deficiency rats. We determined that the learning and memory damage in Kidney-yang deficiency due to synaptic plasticity impairment and increases of A**β**_40_ and 42 was not caused via NMDA receptor internalization induced by A**β** increase. **β**-Adrenergic receptor agonist can rescue the impaired long-term potential (LTP) in Kidney-yang rats. Taken together, our results suggest that spatial learning and memory inhibited in Kidney-yang deficiency might be induced by A**β** increase and the decrease of **β**_2_ receptor function in glia.

## 1. Introduction

Alzheimer's disease (AD), the most common cause of dementia, is a chronic disorder characterized by a progressive decline in cognitive function. Great lines of evidence have verified that the formation of AD is a complicated process. The best-known hypothesis to explain AD is that which involves the role of the accumulation of amyloid-*β* (A*β*) peptide in the brain. As one of major pathological hallmarks, A*β* was considered as primary cause [1]. A*β* is generated from A*β* precursor protein (APP) via sequential cleavages by *β*- and *γ*-secretases [2]. Normally A*β* is physiological product. *β*-Secretase binds to N-terminal of A*β* at extracellular domain of APP and *γ*-secretases binds to C terminal of transmembrane domain. The *γ*-secretase is pivotal, because it determines the ratio of two main A*β* species (A*β*_40_ and A*β*_42_) [3], and mutations in its catalytic subunit presenilin-1 (PS1) account for most cases of familial Alzheimer's disease (FAD) [4]. Under pathological condition, A*β*_40_ or A*β*_42_ appears to be the major species in the initial parenchymal deposition [5].

 Although Alzheimer's disease, as a kind of neurodegenerative disease, was not mentioned in Chinese medicine, the symptoms like learning and memory impairment, dementia, and so forth often appeared in the Traditional Chinese Medicine (TCM) theory. In Chinese medicine, kidney plays an essential role in the pathology of senile dementia. Alzheimer's disease was considered as the consequence produced by deficiency of Kidney essence [6–13]. Therefore, in the clinical treatment and research, tonic kidney herbs were applied as the first choice. However, the published results show that the most studies focus on the alleviation of symptom. Because the animal models lack “common behavior,” the nonconsistent or even controversy reports are often published on the different journals. Furthermore, previous investigations which centered on the relationship between dementia and ZHENG (TCM syndrome) were only limited to the symptom improvement, and the mechanism underlying the ZHENG still remains elusive. Kidney-yang deficiency syndrome (KDS) is one of the primary concepts in TCM. Here we used the older rats which showed the features of Kidney-yang deficiency including profuse urination at night, blur hair, long voiding of clear urine, and low basal metabolic rate as the Alzheimer's disease model and tried to identify the relationship between Kidney-yang deficiency and senile dementia and the underlying mechanism.

## 2. Materials and Methods

### 2.1. Animals

 Male Sprague Dawley (350–400 g) rats were purchased from the Shandong Laboratory animals Center. In this study, all manipulations and procedures were carried out in accordance with The Guide for Care and Use of Laboratory Animals issued by USA National Institutes of Health and were approved by the Animal Care and Use Committee of Shandong University of Traditional Chinese Medicine. As described previously [14, 15], rats were housed (23 ± 1°C) in groups and maintained under a 12-hour light/dark cycle with food and water available *ad libitum*. The rats with same age whose basal metabolic rate (BMR) was 15% lower than normal value, locomotor activity decreased, fur was blur, and urine was 40% more than normal volume were selected as Kidney-yang deficiency model otherwise, the rats were used as control.

As described previously [16], BMRs were measured with Kalabukhov-skvortsov respirometer. Briefly, the temperature was controlled by water bath (±1°C). KOH was used to absorb CO_2_ which the rat produced, and dry silica gel was used to absorb water. After fasting for 4 h, the rest of BMR was measured. Before and after measurement, individual weight (±0.1 g) and anal temperature (±1°C) were measured. The procedure lasted 40 min, and the value was recorded with a 5 min interval. The chamber temperature was 30°C. The average BMR was shown in Supplementary Figure 1 (see Supplementary Material available online at doi: 10.1155/2012/132829).

The locomotor activity was detected in open-field chamber (91.4 × 91.4 cm). Rats were allowed to freely explore the testing chamber for 5 minutes while their distance and jumping activity were recorded through a video which mounted at the above of the chamber. In addition, we also analyzed the time in different zones. The locomotor activity results were shown in Supplementary Figure 2.

### 2.2. Morris Water Maze Test

Morris water maze test was performed as described [17–20]. A circular, black painted pool (150 cm diameter, 50 cm height) filled to a depth of 35 cm with water was used. The water was maintained at 20 ± 1°C and made opaque by the addition of 30 mL of black ink. The pool was divided into four quadrants with four starting locations called north (N), east (E), south (S), and west (W) at equal distance on the rim. An invisible black platform (10 cm diameter) was submerged 1.5 cm below the water line and placed in the center of the northeast quadrant. Rats were trained and tested for 5 days. The rats were trained in the water maze to find and escape onto the hidden platform with a 120 sec cutoff time. Each rat was gently placed into the water, with the nose pointing toward the wall at one of the starting points. The escape latency, the time required for the rats to climb onto the platform, was recorded as the average of four trials. The searching patterns of animals were also recorded when the platform was removed from the pool on day 6. 

### 2.3. Electrophysiology

*In vivo *recording of field excitatory postsynaptic potential (fEPSP) was made from the CA1 stratum radiatum of the right hippocampal hemisphere in response to stimulation of the Schaffer collateral-commissural pathway. The electrode was implanted in male Sprague Dawley rats as described previously [19–23]. Briefly, the surgery was carried out under deep urethane (1.5 mg/kg, intraperitoneally) anesthesia. Two small burr holes (1.5 mm diameter) were drilled in the skull for placing the recording electrode and bipolar stimulating electrode. The recording electrode was inserted 3.4 mm posterior to bregma and 2.5 mm right of the midline. The bipolar stimulating electrode was inserted 4.2 mm posterior to bregma and 3.8 mm right of the midline. The electrodes were lowered slowly through the cortex to a depth of 2.5 mm, the final depths were adjusted until the appearance of a negative deflecting excitatory postsynaptic potential (EPSP), then fixed to the bone with acrylic dental cement. The right placement of electrodes in the stratum radiatum of the CA1 region of the dorsal hippocampus was verified by postmortem examination. The recording and stimulating electrodes (0.1 mm diameter) were made by stainless steel needles (0.1 mm) coated with Teflon.

Recording was performed 2 weeks later in freely moving rats after their recovery from surgery. In all experiments, test fEPSP was evoked by stimulating with a square-wave constant current pulse of 50 *μ*s duration at a frequency of 0.033 Hz. At the beginning of each experiment, input-output curves (stimulus intensity versus fEPSP slope) were generated to determine the maximal fEPSP slope, and then the intensity of stimulus was set at a level that evoked an fEPSP slope of 50–60% of the maximum. The slope of fEPSP was measured. LTP was induced by high-frequency stimulation (HFS) using 20 pulses at 200 Hz, repeated three times at a 30 sec interval. Stimuli were delivered from an isolator connected with Stimulator (Nihon Kohden, Tokyo, Japan). All recording was performed using Pclamp 10.1 (Molecular Devices, Sunnyvale, USA). Two consecutive sweeps were averaged.

*In vitro *acute hippocampal slices from male Sprague Dawley rats were prepared as previously described [20, 24, 25]. Briefly, the slices (400 *μ*m thickness), which were cut acutely in iced and 95% O_2_/5% CO_2_ oxygenated cutting medium including (mM) 230 sucrose, 2.5 KCl, 10 MgSO_4_, 1.25 Na_2_HPO_4_, 26 NaHCO_3_, 0.5 CaCl_2_, 10 D-glucose, were incubated more than 1 h in the artificial cerebrospinal fluid (ACSF) saturated with 95% O_2_/5% CO_2_ at 23 ± 1°C. The ACSF contains (in mM) 124 NaCl, 5 KCl, 2.5 CaCl_2_, 1.3 MgSO_4_, 1.2 KH_2_PO_4_, 26 NaHCO_3_, and 10 Glucose. During recording, the slices were continuously superfused with oxygen-saturated ACSF at room temperature (23 ± 1°C). fEPSPs were recorded using Pclamp 10.1 by placing a glass pipette (3–5 MΩ) filled with NaCl (4 M) in the stratum radiatum of the CA1 region of the hippocampus 100–150 *μ*m away from the cell body layer. Stimuli (200 *μ*s pulse duration) were delivered at 0.017 Hz through a bipolar platinum electrode placed at the level of the Schaffer collaterals from CA3. The response curves evoked by the test stimulus pulse eliciting 50–60% of a maximum fEPSP slope were recorded for 15 min, and LTP was induced with the same stimulating strength by a train of 100 pulses at 100 Hz. One episode of HFS was used. Slices displaying an unstable baseline recording were discarded. All the recordings in hippocampal slices were done at room temperature.

### 2.4. ELISA for A*β*

As described previously [26], we examined A*β*_40_ and A*β*_42_ with sandwich ELISA kits (BioSource, Grand Island, USA). Rat hippocampus was homogenized and centrifuged at 100,000 g for 1 h. We detected rat A*β*_40_ and A*β*_42_ in supernatants with BNT77/BA27 and BNT77/BC05 sandwich ELISA kits (Wako, San Diego, USA) according to previous reports [27]. All measurements were performed in duplicate.

### 2.5. Fluorogenic Substrate Assay

We performed the assay as reported [26]. After centrifugation of tissue homogenate aliquots at 13,000 g for 15 min, pellets were resuspended and incubated at 37°C for 2 h in 50 *μ*L of assay buffer (pH 6.5) containing 12 mM fluorogenic substrates (Calbiochem, Philadelphia, USA). The fluorescence was measured using SpectraMax M5 spectrometer (Molecular Devices) with the excitation wavelength set at 355 nm and the emission wavelength set at 440 nm.

### 2.6. Surface Protein Cross-Linking Assay

 As described previously [28, 29], The cell membrane impermeable cross-linker bis(sulfosuccinimidyl) suberate (BS^3^) (Pierce, Rockford, USA) was applied to examine internalization of surface proteins. Immediately after cutting hippocampal slices acutely, BS^3^ (1 mg/mL) was applied for 40 min at 4°C to link all proteins on the neuronal surface. A thorough wash with PBS was made to remove free BS^3^, and then the tissues were homogenized, lysed, and subjected to SDS-PAGE for Western blot analysis to detect proteins which were not on cell surface. Lysates of cells without BS^3^ treatment and cytosolic proteins such as actin were probed as controls.

### 2.7. Data Analysis

All data is expressed as mean ± SEM. Sigma plot 9.0 (Systat Software Inc., Northampton, USA) and SAS software package (Release 6.12, Sas Institute Inc., Cary, USA) was used to plot and analyze data by unpaired *t*-test for two groups, two-way analysis of variance (ANOVA). *P* < 0.05 was considered statistically significant.

## 3. Results

### 3.1. Spatial Learning Was Impaired in Kidney-Yang Deficiency Rats

**Figure 1:**
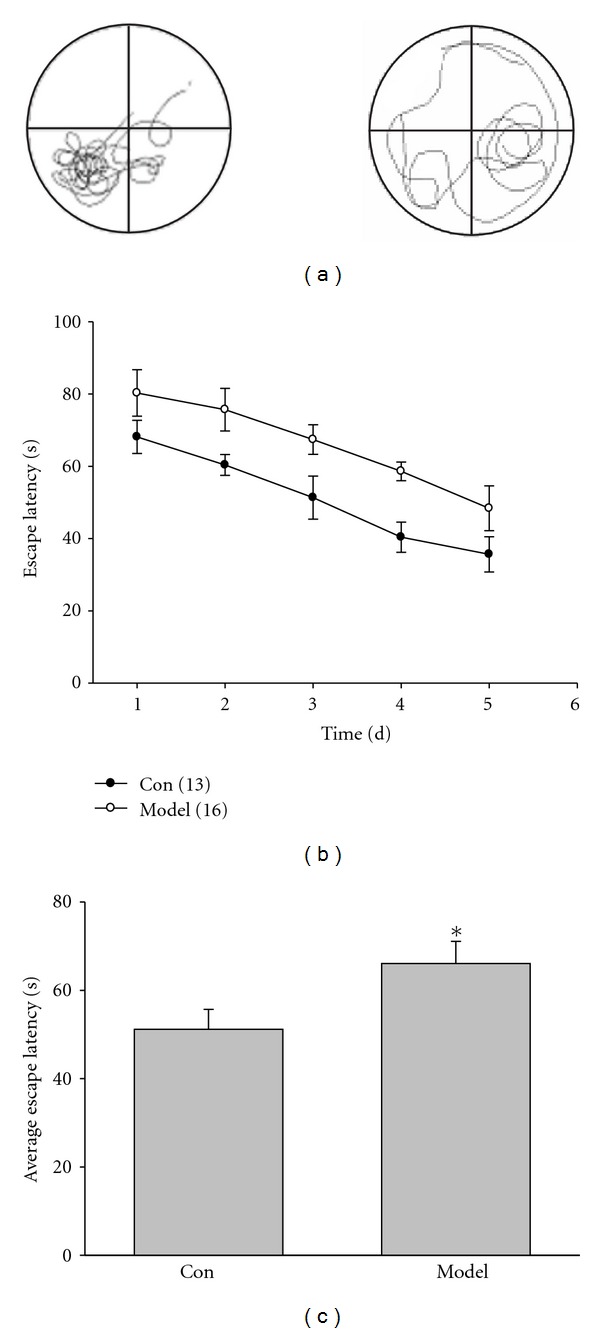
Rats were trained and tested in the Morris water maze with platform hidden at the third quadrant. (a) Representative swimming traces in the Morris water maze. (b) The latency to find the hidden platform at different time points. (c) The average latency of 5 days. Kidney-yang deficiency rats decreased the escape latency significantly, compared to the control (*P < 0.05, unpaired Student's *t*-test); *n* = 13 for control, 16 for model.

In the present study, we first investigated the learning and memory of rats in Morris water maze. One week after finishing BMR measurement, the rats were trained in Morris water maze for 5 days. As shown in Figures 1(a) and 1(b), the escape latency for searching hidden platform of model rats was longer than that of control at different time points.  Figure 1(c) showed that the average escape latency of model rats increased significantly compared to control (model: 66.06 ± 5.04 s, *n* = 13; control: 51.17 ± 4.50 s, *n* = 16; *P* < 0.05).

**Figure 2:**
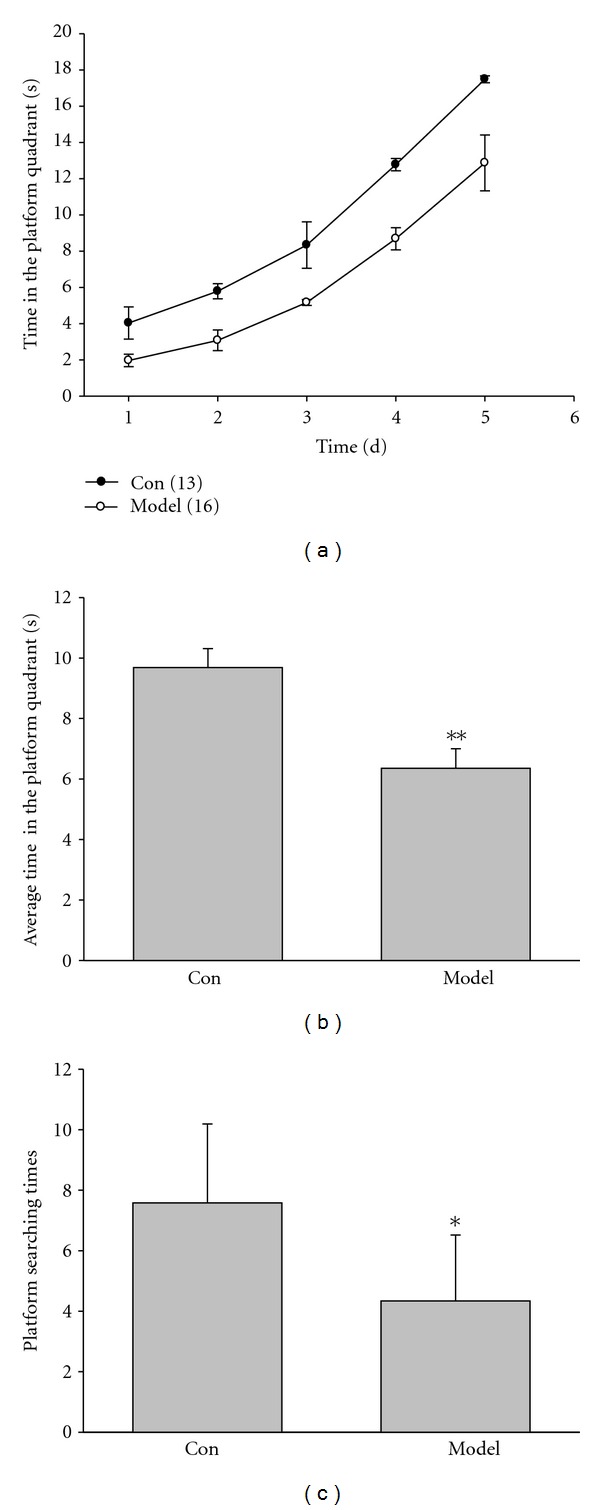
Rats were trained and tested in the Morris water maze without platform. (a) The time spent in the platform quadrant at different time points. (b) The average time spent in the platform quadrant for 5 days. Kidney-yang deficiency rats decreased the time spent in the platform quadrant significantly, compared to the control (*P < 0.01, unpaired Student's *t*-test). (c) At the fifth day, average entering times to the platform quadrant. The times when Kidney-yang deficiency rats entered platform quadrant after removing the platform decreased significantly (*P* < 0.05, unpaired Student's *t*-test). *n* = 13 for control, 16 for model.

In the test in which the platform was removed, the model rats stayed less time in this quadrant than control group did. As shown in Figures 2(a) and 2(b), the performance of the model rats was poorer than that of control (control: 9.69 ± 0.62 s, *n* = 13; model: 6.36 ± 0.64 s, *n* = 16; *P* < 0.01). At the fifth day, average entering times reduced significantly (control: 7.58 ± 2.61, *n* = 13; model: 4.34 ± 2.18, *n* = 16; *P* < 0.05.  Figure 2(c)), suggesting that Kidney-yang deficiency can damage the spatial learning and memory capacity in rats.

### 3.2. Swimming Capacity Was Damaged in Kidney-Yang Deficiency Rats

**Figure 3:**
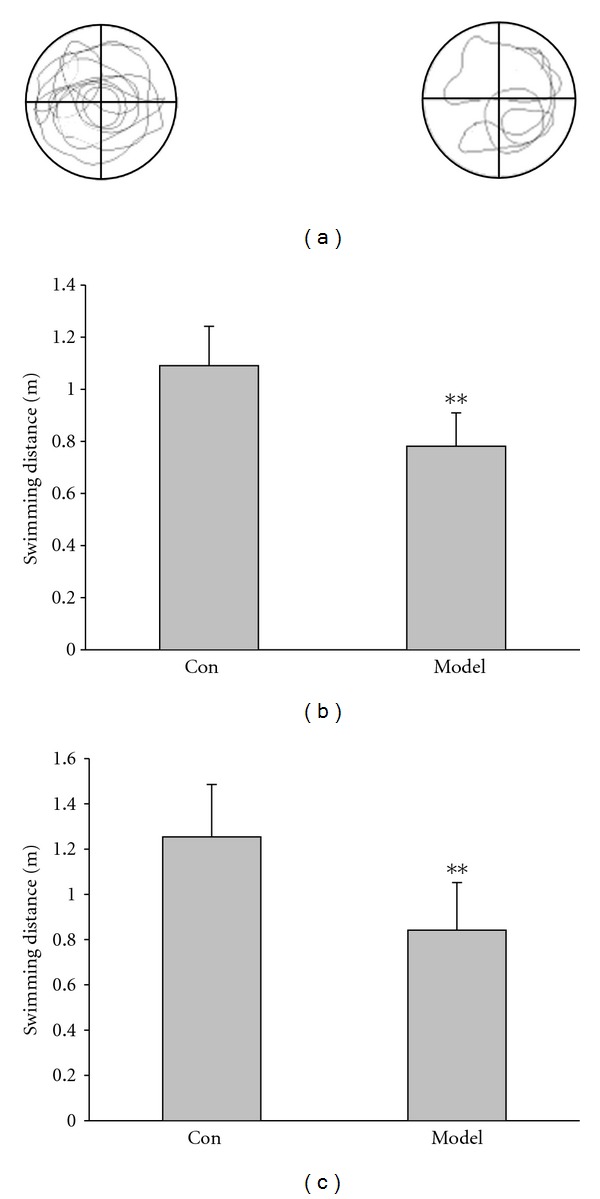
Swimming capacity was detected in the Morris water maze. (a) Representative swimming traces in the Morris water maze after removing the platform. (b) With the hidden platform in the third quadrant, and the average swimming distance of model rats is shorter than that of the control (**P < 0.01, unpaired Student's *t*-test). (c) No platform in the third quadrant, and the average swimming distance of model rats is also shorter than that of the control (**P < 0.01, unpaired Student's *t* test). *n* = 13 for control, 16 for model.

In Morris water maze, we also detected the swimming capacity. As shown in Figures 3(a) and 3(b), with the hidden platform in the third quadrant, the average swimming distance of model rats is shorter than that of the control (control: 1.09 ± 0.15 m, model: 0.78 ± 0.13 m; *P* < 0.0, *n* = 16).  Figure 3(c) shows the similar results after removing the platform (control: 1.25 ± 0.23 m, model: 0.84 ± 0.21 m; *P* < 0.01, *n* = 16). These results suggested that the motor capacity was damaged by Kidney-yang deficiency. 

### 3.3. A*β*_40_ Increased in Kidney-Yang Deficiency Rats

**Figure 4:**
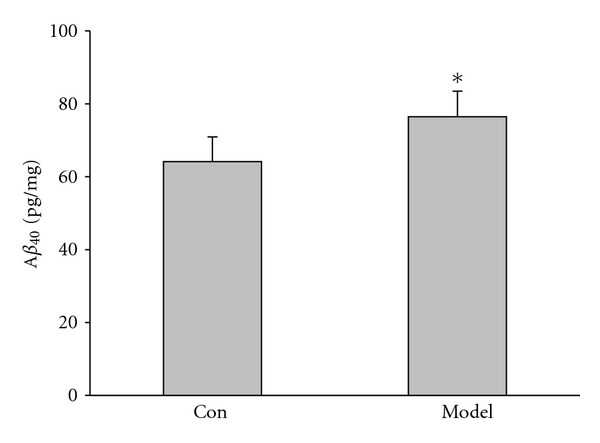
ELISA for A*β*_40_ in the hippocampus. ELISA shows the secreted A*β*_40_ increased significantly in the hippocampus from the Kidney-yang deficiency (*P < 0.05, unpaired Student's *t*-test).

Previous investigations demonstrated that Amyloid plaque which is largely composed of A*β* in brain is one of the typical pathological characteristics of Alzheimer's disease [30, 31]. A*β* with 40 or 42 amino acid sequences can accumulate easily. Here we thus detected the expression of A*β*_40_ in hippocampus of Kidney-yang deficiency rats. As shown in Figure 4, the expression of A*β*_40_ in model rats (76.43 ± 4.03 pg/mg) is increased significantly compared to that of the control (64.13 ± 6.76 pg/mg, *P* < 0.05).

### 3.4. A*β*_42_ Was Increased in Kidney-Yang Deficiency Rats

**Figure 5:**
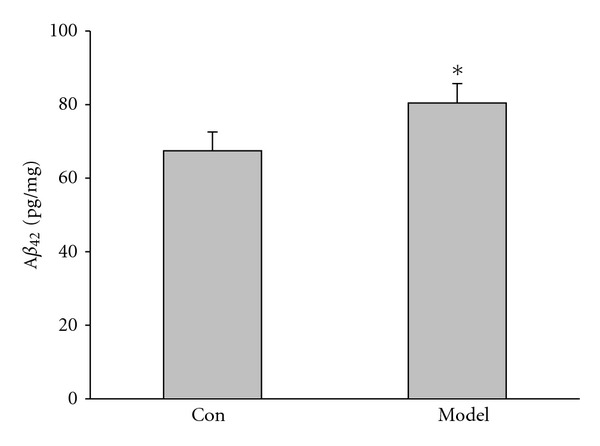
ELISA for A*β*_42_ in the hippocampus. ELISA shows the secreted A*β*_42_ increased significantly in the hippocampus. from the Kidney-yang deficiency (*P < 0.05, unpaired Student's *t* test).

Here we also investigated the expression of A*β*_42_ in hippocampus. As shown in Figure 5, A*β*_42_ increased significantly in rat hippocampus, compared to the control (model: 80.45 ± 5.28 pg/mg; 67.43 ± 5.12 pg/mg, *P* < 0.05). The above results suggested that hippocampal A*β* increase could contribute to the learning and memory impairment of Kidney-yang deficiency rats.

### 3.5. Activity of Hippocampal *γ*-Secretase Was Not Changed in Kidney-Yang Deficiency Rats

**Figure 6:**
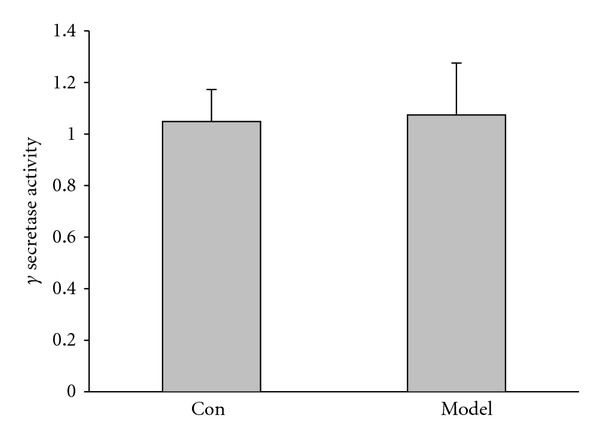
Determination of *γ*-secretase activity using a Fluorogenic substrate assay. *γ*-Secretase activity of hippocampus from Kidney-yang deficiency rats did not changed significantly (*P* > 0.05, unpaired Student's *t* test), *n* = 3 for both groups.

Subsequently a question was raised: what causes hippocampal A*β* increase in Kidney-yang deficiency? Previous reports showed that A*β* is generated from A*β* precursor protein (APP) via sequential cleavages by *β*- and *γ*-secretases [2]. However, the *γ*-secretase is pivotal, because it determines the ratio of two main A*β* species (A*β*_40_ and A*β*_42_) [3]. Here we used Fluorogenic substrate assay to examine the activity of hippocampal *γ*-secretase.  Figure 6 showed that the activity of hippocampal *γ*-secretase did not change significantly, compared to the control (model: 1.07 ± 0.20; control: 1.05 ± 0.12; *P* > 0.05), suggesting that the increase of hippocampal A*β* expression is not caused by upregulation of *γ*-secretase in Kidney-yang deficiency rats.

### 3.6. Learning and Memory Impairment of Kidney-Yang Deficiency is Not Caused by Internalization of Hippocampal NMDA Receptors

**Figure 7:**
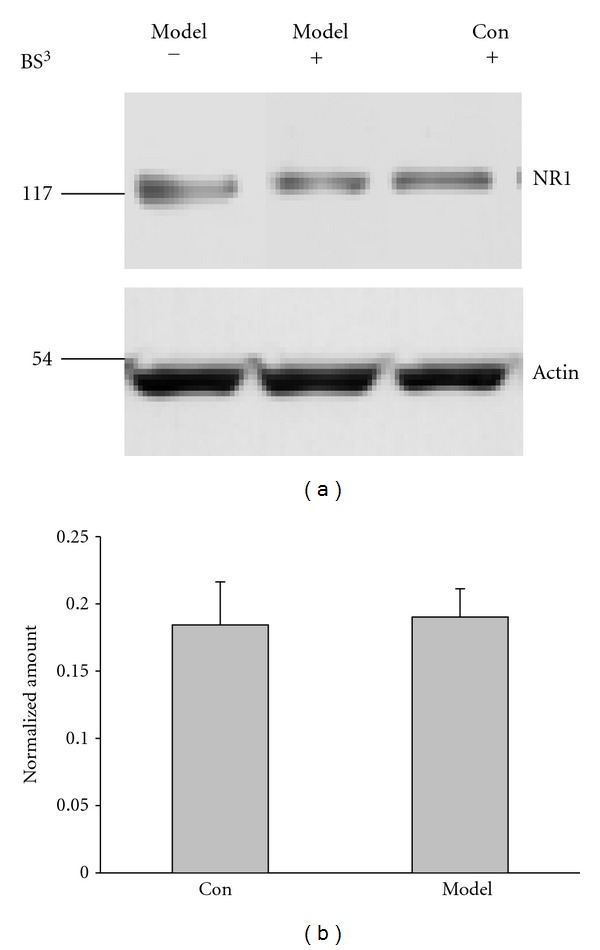
Surface protein biotinylation assay for NMDA receptors (NMDARs) of hippocampal neuron membrane. (a) Representative Western blotting. (b) Summarized data from 4 trials shows that NMDARs expressed on the neuron surface did not change significantly compared to the control (*P* > 0.05, unpaired Student's *t*-test), *n* = 3 for control, 5 for model.

It is well documented that A*β* increase could cause NMDA receptor (NMDAR) internalization which is involved in Alzheimer's disease [32]. To determine the role of NMDAR internalization, we used surface protein biotinylation assay to analyze the expression of NMDAR of hippocampal neuron membrane. As shown in Figures 7(a) and 7(b), neuron surface NMDAR expression did not show significant difference between the model and the control (control: 0.18 ± 0.03 model: 0.19 ± 0.02; *P* > 0.05), suggesting that NMDAR internalization did not contribute to the learning impairment of Kidney-yang deficiency.

### 3.7. Damage of Synaptic Plasticity Is Involved in the Learning and Memory Impairment of Kidney-Yang Deficiency Rats

**Figure 8:**
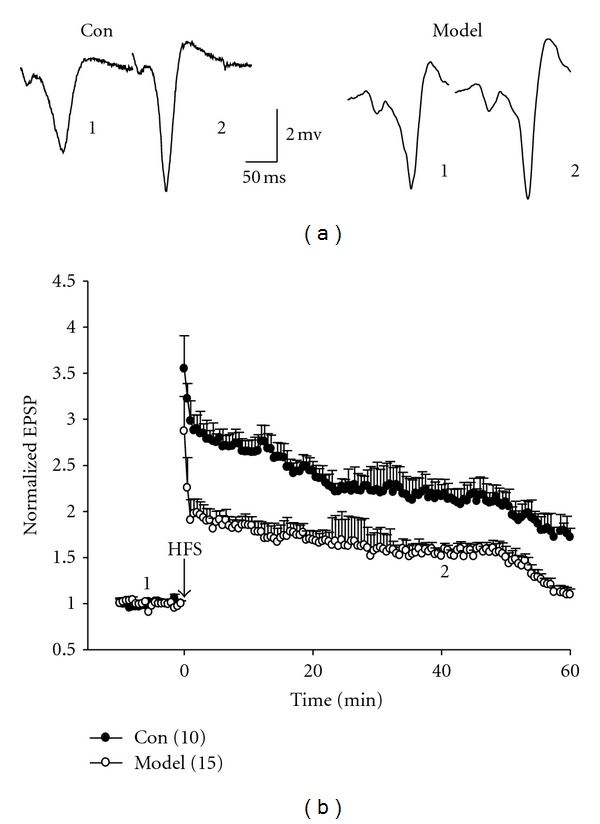
Long-term potential (LTP) recorded *in vivo.* (a) Representative sweeps of field excitatory postsynaptic potential (fEPSP) recorded in the freely moving rats. (b) Summary of averaged normalized fEPSP slope from (a). Compared to the control, the LTP was significantly inhibited in Kidney-yang deficiency rats (*P* < 0.05, two-way ANOVA), *n* = 10 for control, 14 for model. “1” and “2” in (a) and (b) indicate the sweeps recorded separately at 5 min and 40 min before and after high-frequency stimulation.

 Previous studies addressed that long-term potentiation (LTP) is associated with learning and memory [33, 34]. A*β* increase can damage hippocampal synaptic plasticity. Here we used extracellular recording *in vivo* to investigate change of the LTP. As shown in Figures 8(a) and 8(b), after high-frequency stimulation (HFS), the LTP was reduced significantly in model than that of the control (*P* < 0.05), suggesting that damage of hippocampal synaptic plasticity could be one of mechanisms responsible for learning and memory impairment.

### 3.8. *β*-Adrenergic Receptor Agonist Can Alleviate the Impairment of LTP in Kidney-Yang Rats

**Figure 9:**
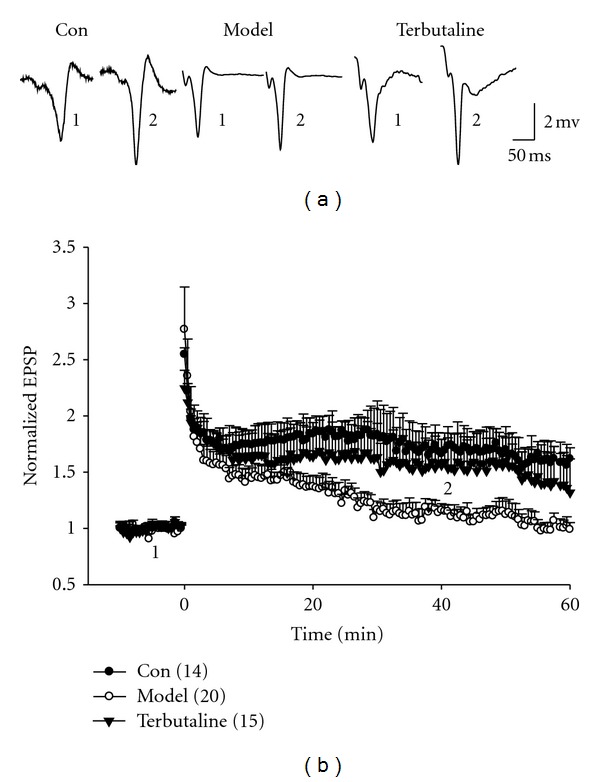
Long-term potential (LTP) recorded in acute hippocampal slices. (a) Representative sweeps of field excitatory postsynaptic potential (fEPSP) recorded in acute hippocampal slices. (b) Summary of averaged normalized fEPSP slope from (a). Compared to the control, the LTP was significantly inhibited in Kidney-yang deficiency rats, but can be rescued by terbutaline, a *β*_2_ receptor agonist (*P* < 0.05, two-way ANOVA), *n* = 10 for control, 14 for model, 11 for terbutaline. “1” and “2” in (a) and (b) indicate the sweeps recorded separately at 5 min and 40 min before and after high-frequency stimulation.

*β*-Adrenergic receptor (*β*_2_-AR) is expressed in the hippocampus and cortex. Activating *β*_2_-AR can enhance the activity of *γ*-secretase and thus cause an increase in A*β* production. However, as shown in Figure 6, the activity of *γ*-secretase did not change significantly, suggesting that A*β* expression increase is not caused by upregulating activity of *γ*-secretase. Here we examine whether *β*_2_-AR activity can affect the hippocampal LTP of Kidney-yang deficiency through recording LTP in acute brain slices. Figures 9(a) and 9(b) showed that the LTP was inhibited in the brain slices from the Kidney-yang deficiency rats (*P* < 0.01), consistent with the *in vivo* recording; specific *β*_2_-AR agonist terbutaline can significantly improve the LTP of the acute hippocampal slice from the model rats (*P* < 0.05), suggesting that glia *β*_2_-AR dysfunction may contribute to the inhibition of LTP in the Kidney-yang deficiency rats.

## 4. Discussion

In the present study, we firstly selected the Kidney-yang deficiency rats through evaluating the locomotors activity, BMR, total urine volume of 24 h combining with the fur, and demonstrated that spatial learning and escape capacity were significantly impaired in Kidney-yang deficiency rats. We also found that A*β* with 40 and 42 amino acid sequences increased expression in hippocampus of Kidney-yang deficiency rats, consistent with the previous reports in which A*β* enhanced in brain is one of the typical pathological characteristics of Alzheimer's disease [1]. Although the *γ*-secretase is pivotal in determining the ratio of two main A*β* species (A*β*_40_ and A*β*_42_), but we demonstrated in Kidney-yang deficiency rats, *γ*-secretase activity did not change significantly. As previous reports [32], NMDA receptor (NMDAR) internalization induced by hippocampal A*β* increase is involved in Alzheimer's disease; however, the result that neuron surface NMDAR expression did not show significant reduction in Kidney-yang deficiency rules out the role of NMDAR internalization in the learning and memory impairment of Kidney-yang deficiency. The damage of synaptic plasticity caused by A*β* increase could be rescued by *β*_2_-AR agonist. Therefore, our studies firstly address that the increase of A*β* may contribute to the learning and memory impairment of Kidney-yang deficiency rats, and *β*_2_-AR inhibition plays an important role in the hippocampal A*β* increase in this model.

Previous studies showed that stress-activated *β*-ARs not only regulate the secondary message level and subsequently affect the signal transduction but also play a role in receptor internalization which is associated with the receptor desensitization and signal transduction mediated by clathrin [35, 36]. *β*_2_-ARs express highly in the cortex and hippocampus [37]. The previous reports demonstrated that the activation of *β*_2_-AR promotes the activity of *γ*-secretase and thus increase A*β*. *In vivo* experiments in AD transgenic mice also verified that after treating with *β*_2_-AR agonist isoproterenol for a long time, the A*β* plaque enhanced in the mouse brain and on the contrary antagonist ICI118551 reduced the plaque [26]. However, the current study shows that although A*β* expression increases significantly in Kidney-yang deficiency rats, the activity of *γ*-secretase did not change significantly, suggesting that the A*β* increase may not be due to the enhancement of the activity of *γ*-secretase by overactivating *β*_2_-AR.

 As mentioned above, A*β* can affect the neuron surface receptors, NMDAR is one of the affected receptors [38]. Our studies demonstrated that the neuron surface receptors were not internalized in Kidney-yang deficiency rats. Interestingly, the damaged LTP is involved in the decrease of learning capacity of Kidney-yang deficiency. LTP in acute brain slices showed that *β*_2_-AR agonist can ameliorate the inhibited LTP. However, the question is: What causes A*β* increase in the condition without *β*_2_-AR increase or overactivity? How does A*β* inhibit the LTP? As well known, glia expresses a lot of *β*_1_ and *β*_2_ receptors, and *β*_2_ receptors is primary. Previous investigation demonstrated that LTP could be damaged by increasing TNF release induced by A*β* enhancement [39]. The brain-derived TNF is produced by glia and could be inhibited by *β*_2_ receptor agonist.

## 5. Conclusions

 Taken together, our studies indicated that the spatial learning inhibited in kidney-yang deficiency might be due to hippocampal synaptic plasticity damaged by A*β*_40_ and 42 increase which are associated with the decrease of *β*_2_ receptor function in glia.

##  Authors' Contribution

D. Qi and Y. Qiao are equally contributed to this work. 

## Supplementary Material

Supplementary Figure 1: Basal metabolic rate (BMR) and Urine volume of 24 hours in Kidney-yang deficiency rats. (a) Averaged BMR. BMRs were measured with Kalabukhov-skvortsov respirometer. (b) Urine volume for 24 hours. ∗ p<0.05, unpaired student's t test; n=51 for control, 63 for model.
Supplementary Figure 2: Locomotor activity took place in the Open field chamber. (a) Moving distance measured for 5 minutes. (b) The averaged jumping times of 5 minutes. (c) Staying time at the board zone of the chamber. The duration which the Kidney-yang deficiency rats stayed at the board zone was increased significantly (∗∗ p<0.01, student's t test). (d) For Kidney-yang deficiency rats, the time of staying at center zone of the chamber decreased significantly (∗∗ p<0.01, unpaired student's t test); n=51 for control, 63 for model.
