# Supplementary material for: Aβ Damages Learning and Memory in Alzheimer's Disease Rats with Kidney-Yang Deficiency
Source: Evid Based Complement Alternat Med. 2012 May 8;2012:132829. doi: 10.1155/2012/132829 (PMC3356918; doi:10.1155/2012/132829)
Supplement: Supplementary file 1 — Supplementary Figure 1: Basal metabolic rate (BMR) and Urine volume of 24 hours in Kidney-yang deficiency rats. (a) Averaged BMR. BMRs were measured with Kalabukhov-skvortsov respirometer. (b) Urine volume for 24 hours. ∗ p<0.05, unpaired student's t test; n=51 for control, 63 for model. Supplementary Figure 2: Locomotor activity took place in the Open field chamber. (a) Moving distance measured for 5 minutes. (b) The averaged jumping times of 5 minutes. (c) Staying time at the board zone of the chamber. The duration which the Kidney-yang deficiency rats stayed at the board zone was increased significantly (∗∗ p<0.01, student's t test). (d) For Kidney-yang deficiency rats, the time of staying at center zone of the chamber decreased significantly (∗∗ p<0.01, unpaired student's t test); n=51 for control, 63 for model. [file 132829.f1.doc]

**Supplementary figure 1 Basal metabolic rate (BMR) and Urine volume of 24 hours in Kidney-yang deficiency rats**

(a) BMR. (b) Urine volume for 24 hours. * p<0.05, unpaired student’s t test; n=51 for control, 63 for model.

**Supplementary figure 2 Locomotor activity took place in the Open field chamber**

(a) Moving distance measured for 5 minutes. (b) The averaged jumping times of 5 minutes. (c) Staying time at the board zone of the chamber. The duration which the Kidney-yang deficiency rats stayed at the board zone was increased significantly (** p<0.01, student’s t test). (d) For Kidney-yang deficiency rats, the time of staying at center zone of the chamber decreased significantly (** p<0.01, unpaired student’s t test); n=51 for control, 63 for model.

Supplementary fig. 1

a

b

Fig. 1 BMR and urine volume

Supplementary fig. 2

a

b

c

d
